# The Structure-Activity Relationship of Pterostilbene Against *Candida albicans* Biofilms

**DOI:** 10.3390/molecules22030360

**Published:** 2017-02-27

**Authors:** Dan-Dan Hu, Ri-Li Zhang, Yong Zou, Hua Zhong, En-Sheng Zhang, Xiang Luo, Yan Wang, Yuan-Ying Jiang

**Affiliations:** 1 New Drug Research and Development Center, School of Pharmacy, Second Military Medical University, Shanghai 200433, China; dandanhu1989@163.com (D.-D.H.); zhangrili116@163.com (R.-L.Z.); zhonghuasmmu@hotmail.com (H.Z.); 2 School of Pharmaceutical Sciences, Sun Yat-sen University, Guangzhou 510006, China; zou_jinan@163.com (Y.Z.); rosen_luo@163.com (X.L.); 3 School of Chemistry and Chemical Engineering, Yan’an University, Yan’an 716000, China; sdzes2006@163.com; 4 ZhongshanWanYuan New Drug R & D Co., Ltd., Zhongshan 528451, China

**Keywords:** *Candida albicans*, pterostilbene, anti-biofilm, structure-activity relationship

## Abstract

*Candida albicans* biofilms contribute to invasive infections and dramatic drug resistance, and anti-biofilm agents are urgently needed in the clinic. Pterostilbene (PTE) is a natural plant product with potentials to be developed as an anti-biofilm agent. In this study, we evaluated the structure-activity relationship (SAR) of PTE analogues against *C. albicans* biofilms. XTT (Sodium 2,3-bis(2-methoxy-4-nitro-5-sulfophenyl)-2*H*-tetrazolium-5-carboxanilide inner salt) reduction assay was used to evaluate the activity of the analogues against *C. albicans* biofilms. Knowing that hyphal formation is essential for *C. albicans* biofilms, anti-hyphal assay was further carried out. By comparing a series of compounds tested in this study, we found that compounds with *para*-hydroxy (–OH) in partition A exhibited better activity than those with other substituents in the *para* position, and the double bond in partition B and *meta*-dimethoxy (–OCH_3_) in partition C both contributed to the best activity. Consistent results were obtained by anti-hyphal assay. Collectively, *para*-hydroxy (–OH), double bond and *meta*-dimethoxy (–OCH_3_) are all needed for the best activity of PTE against *C. albicans* biofilms.

## 1. Introduction

*Candida albicans* is one of the most common human fungal pathogens. It can cause superficial to life-threatening infections, especially in immune-compromised patients. Notably, *C. albicans* can develop biofilms on the surface of medical implant devices [1,2]. Biofilms are microbial communities with three-dimensional structure [3], and hyphae are essential for *C. albicans* biofilms [4]. The formation of biofilms increased resistance of *C. albicans* to antifungal agents, as well as host immune defenses [5,6]. Additionally, the biofilms on medical devices contribute to repeated infections and may lead to the failure of implant operations [7].

**Figure 1:**
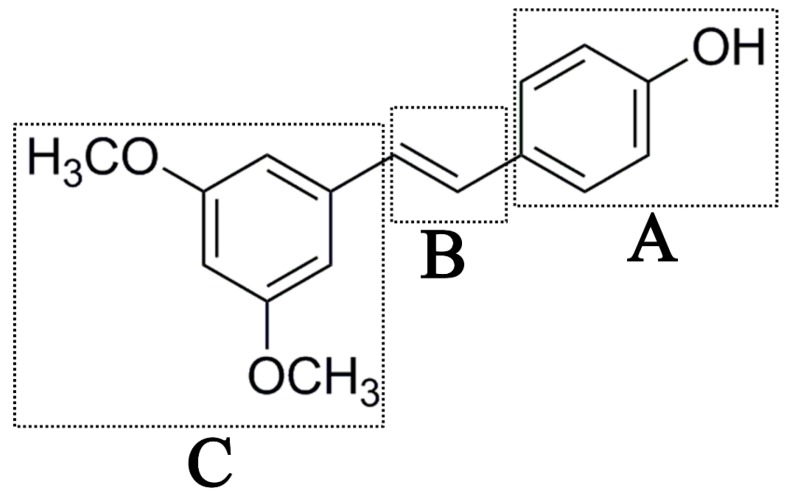
Chemical structures of PTE and three partitions of PTE.

Antifungal drugs with anti-biofilm activity are very limited in the clinic, and anti-biofilm drugs are urgently needed [8]. In our previous study, we found that pterostilbene (PTE) had a strong bioactivity against *C. albicans* biofilms, and might serve as a lead compound to develop anti-biofilm drugs [9]. PTE (Figure 1) is a stilbene-derived phytoalexin that originates from several natural plant sources, such as *Pterocarpusmarsupium* (the Indian kino tree), *Pterocarpus santalinus* (red sandal wood), *Vitis vinifera* (common grape vine), and *Vaccinium ashei* (rabbiteye blueberry) [10]. In this study, we investigated the structure-activity relationship (SAR) of PTE against *C. albicans* biofims.

## 2. Results

### 2.1. In the Para Position of Partition A, Hydroxy (–OH) Takes Advantages over Other Substituents

To better understand the SAR of PTE, we investigated several partitions of PTE. Partition A contains 4-hydroxy benzene, partition B is a double bond, and partition C contains 3,5-dimethoxy benzene (Figure 1).

**Figure 2:**
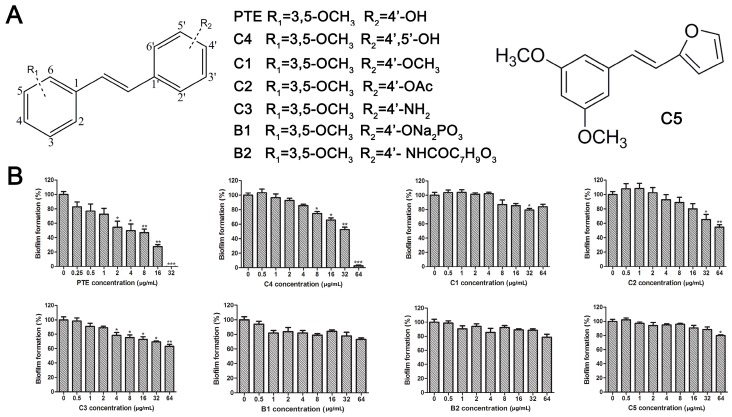
Effects of PTE and analogues C1–C5, B1, B2 on biofilm formation. (**A**) Chemical structures of PTE and analogues C1–C5, B1, and B2 are shown. The structures of analogue C1–C5, B1, and B2 are similar with PTE, and differences only exist in partition A; (**B**) Biofilm formation was evaluated by XTT reduction assay, and the results were presented as the percentage compared to the control biofilms formed without compound treatment. Biofilm formation results represent the mean ± standard deviation fromthree independent experiments. * *p* < 0.05 compared with the control biofilms, ** *p* < 0.01 compared with the control biofilms, *** *p* < 0.001 compared with the control biofilms. Statistical analysis was performed to compare the effects of various compounds, and significant difference was observed upon all the analogues compared with PTE. *p* = 0.035 (C4 vs. PTE), *p* < 0.001 (C1 vs. PTE), *p* < 0.001 (C2 vs. PTE), *p* = 0.0047 (C3 vs. PTE), *p* < 0.001 (B1 vs. PTE), *p* < 0.001 (B2 vs. PTE), and *p* < 0.001 (C5 vs. PTE).

The structures of analogues C1–C5, B1 and B2 are similar with PTE, and differences only exist in partition A. In C4, a hydroxy is added to the 5’ position of partition A. In C1, C2, C3, B1, and B2, 4’-*para*-hydroxy is changed to 4’-*para*-methoxy, 4’-*para*-acetoxy, 4’-*para*-amino, 4’-*para*-disodium phosphate, and 4’-*para*-7-oxabicyclo[2.2.1]heptane-2-carboxylic acid-3-amido, respectively. In C5, a furan ring replaces partition A (Figure 2A).

RPMI 1640 (Gibco, Bethesda, MD, USA) medium is the most commonly used medium in *C. albicans* biofilm studies [11], and was used in this work. In RPMI 1640 medium, PTE exhibited good activity against *C. albicans* biofilm formation. At the concentration as low as 2 μg/mL, PTE significantly inhibited biofilm formation, and its SMIC_80_ was determined at 16 μg/mL (Table 1). C4 also exhibited activity against biofilm formation with the SMIC_80_ being 32–64 μg/mL. In contrast, without *para*-hydroxy, C1, C2, C3, B1, and B2 lost the activityin at all the concentrations tested in this study, from 0.5 μg/mL to 64 μg/mL. Similarly, C5 hardly repressed biofilm formation (Figure 2B). Consistently, PTE exhibited activity against pre-formed mature biofilms with SMIC_80_ being 128 μg/mL. C4 exhibited weaker activity with SMIC_80_ being 512 μg/mL. C1, C2, C3, B1, B2, and C5 had no inhibitory activity on mature biofilm (Table 1). Since only C4 and PTE have *para*-hydroxy, we inferred that *para*-hydroxy (–OH) in partition A took advantages over other substituents.

**Figure 3:**
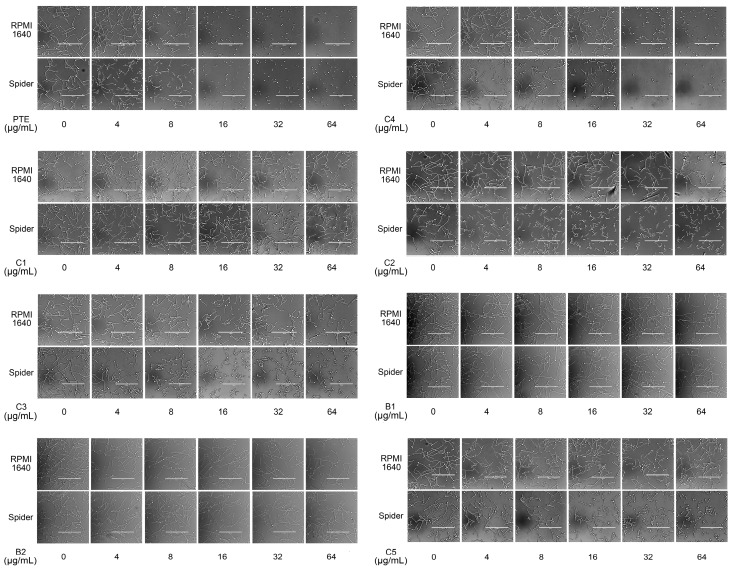
Effects of different concentrations of PTE and analogues C1–C5, B1, and B2 on hyphal formation. Exponentially-growing *C. albicans* SC5314 cells were transferred to hypha-inducing liquid media, including Spider and RPMI1640. The cellular morphology was photographed after incubation at 37 °C for 3 h. Scale bar = 100 μm.

Knowing that hyphal formation is essential for *C. albicans* biofilms, anti-hyphal assay was used to confirm the effects of the compounds on biofilm formation. RPMI 1640 medium and Spider medium are used as hyphal-inducing media, and *C. albicans* can form hyphae in both media (Figure 3). In accordance with the biofilm results, PTE inhibited hyphal formation in both RPMI 1640 and Spider media, and the cells were all yeast-like with 16 μg/mL PTE treatment (Figure 3, Table S2). C4 inhibited hyphal formation in a concentration-dependent manner, and the cells were all yeast-like with 64 μg/mL C4 treatment. C1, C2, C3, B1, and B2 could not inhibit hyphal formation effectively (Figure 3, Table S2). More specifically, most *C. albicans* formed >40 μm hyphae even with 64 μg/mL C1, B1, or B2 in both media. With high concentrations of C2 or C3 (8–64 μg/mL) in Spider medium, most *C. albicans* cells formed pseudohyphae instead of hyphae. In RPMI 1640 medium, about 30%–40% cells formed long hyphae (≥40 μm) even with 64 μg/mL C2 or C3. The effect of C5 on hyphal formation was weak. More specifically, even with 64 μg/mL C5 in RPMI 1640 medium, about 83% cells formed long hyphae (≥40 μm). In Spider medium, the activity of C5 was also weak. Only at the highest concentration tested in this study, 64 μg/mL, C5 affected hyphal formation and most cells formed pseudohyphae (Figure 3, Table S2). Collectively, hyphal formation results were consistent with the findings in biofilm formation assay.

### 2.2. In Partition B, the Double Bond Contributes to the Best Activity

**Figure 4:**
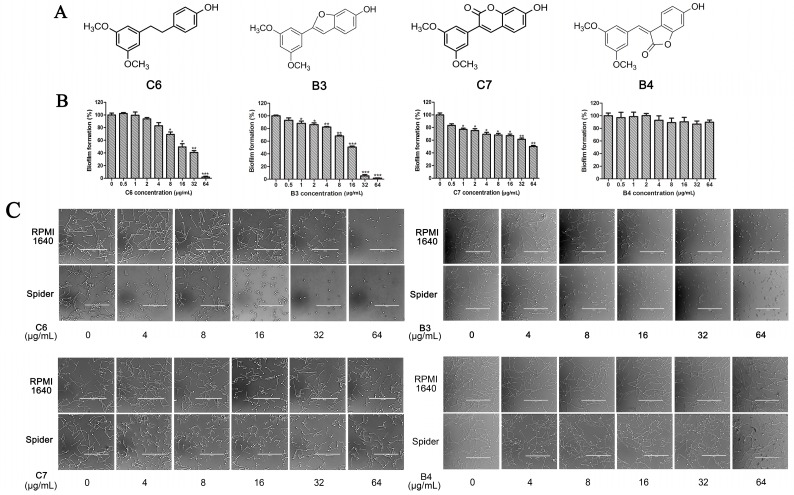
Effects of different concentrations of analogues C6, B3, C7, and B4 on biofilm formation and hyphal formation. (**A**) Chemical structures of analogues C6, B3, C7 and B4 are shown. C6, B3, C7, and B4 are both different from PTE in partition B; (**B**) Effects of different concentrations of analogues C6, B3, C7, and B4 on biofilm formation. Biofilm formation results represent the mean ± standard deviation for three independent experiments. * *p* < 0.05 compared with the control biofilms, ** *p* < 0.01 compared with the control biofilms, *** *p* < 0.001 compared with the control biofilms. Statistical analysis was performed to compare the effects of various compounds, and significant difference was observed upon C6, C7, and B4 compared with PTE. *p* = 0.037 (C6 vs. PTE), *p* = 0.175 (B3 vs. PTE), *p* = 0.0033 (C7 vs. PTE), and *p* < 0.001 (B4 vs. PTE); (**C**) Effects of different concentrations of analogues C6, B3, C7, and B4 on hyphal formation. Scale bar = 100 μm.

Analogues C6, B3, C7, and B4 are different from PTE in partition B. In C6, a single bond replaces the double bond of PTE. In B3 and C7, a furan ring and an unsaturated lactone replaces the double bond of PTE respectively. In B4, the double bond still exists, while partition A and partition B form a cyclic ester (Figure 4A). C6 maintained the activity against biofilm formation, while the activity was a little weaker than PTE. More specifically, 8 μg/mL C6 could inhibit biofilm formation significantly (Figure 4B; *p* < 0.05) and the SMIC_80_ of C6 was 64 μg/mL (Table 1). B3 showed inhibitory activity against biofilm formation almost as strong as PTE, with MIC_80_ being 32 μg/mL. C7 exhibited very weak activity against biofilm formation, and 60% biofilm maintained even at 64 μg/mL. No inhibitory activity was observed of B4 (Figure 4B). Consistent results were obtained in mature biofilm assay. C6 and B3 had a SMIC_80_ of 512 and 128–256 μg/mL, respectively. C7 and B4 showed no obvious inhibitory effect on mature biofiom (Table 1). It can be inferred that the double bond contributed to the best activity of PTE.

In the hyphal formation assay, C6 inhibited hyphal formation in a concentration-dependent manner in both media tested. With 32 μg/mL C6 treatment, 85% cells could not form long hyphae (≥40 μm) in RPMI 1640 medium, and all cells stayed as yeast in Spider medium. B3 was also effective and showed almost the same anti-hyphal activity as PTE. C7 and B4 hardly showed anti-hyphal activity (Figure 4C, Table S2). These results were consistent with the findings in biofilm formation assay.

### 2.3. In Partition C, Meta-Dimethoxy (–OCH_3_) Contributes to the Best Activity

**Figure 5:**
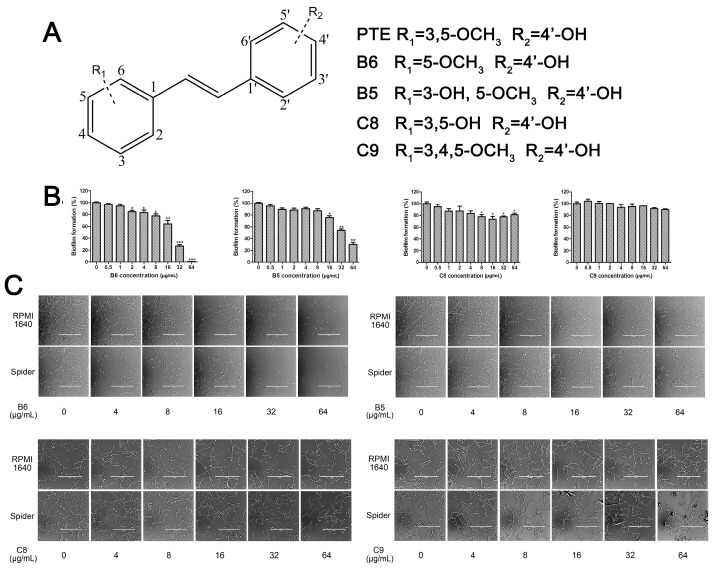
Effects of different concentrations of analogues B6, B5, C8. and C9 on biofilm formation and hyphal formation. (**A**) Chemical structures of analogues B6, B5, C8, and C9 are shown. B6, B5, C8, and C9 are different from PTE in partition C; (**B**) Effects of different concentrations of analogues B6, B5, C8, and C9 on biofilm formation. Biofilm formation results represent the mean ± standard deviation for three independent experiments. * *p* < 0.05 compared with the control biofilms, ** *p* < 0.01 compared with the control biofilms, *** *p* < 0.001 compared with the control biofilms. Statistical analysis was performed to compare the effects of various compounds, and significant difference was observed upon B5, C8, and C9 compared with PTE. *p* = 0.182 (B6 vs. PTE), *p* = 0.041 (B5 vs. PTE), *p* < 0.001 (C8 vs. PTE), and *p* < 0.001 (C9 vs. PTE); (**C**) Effects of different concentrations of analogues B6, B5, C8, and C9 on hyphal formation. Scale bar = 100 μm.

The differences between analogue B6, B5, C8, C9, and PTE only exist in partition C. In B6, only 3-methoxy is reserved. In B5, 3-hydroxy-5-methoxy replaces *meta*-dimethoxy. In C8 and C9, 3,5-*meta*-dimethoxy is changed to 3,5-*meta*-dihydroxy and 3,4,5-trimethoxy, respectively (Figure 5A). B6 showed inhibitory activity against biofilm formation with SMIC_80_ being 64 μg/mL. B5 also showed inhibitory activity at high concentrations, but its activity is significantly weaker than PTE (*p* = 0.041). No obvious inhibitory activity was observed of C8 and C9 (Figure 5B). Consistent results were obtained in investigations on mature biofilms (Table 1). It can be inferred that the compound with *meta*-dimethoxy (–OCH_3_) contributes to the best inhibitory activity.

In accordance with the biofilm results, B6 and B5 obviously inhibited hyphal formation at high concentrations. With 32 μg/L B6 treatment, most *C. albicans* cells were yeast-like. With 64 μg/mL B5 treatment, few cells could form long hyphae (≥40 μm) in media tested in this study. C8 and C9 could not effectively inhibit hyphal formation even at 64 μg/mL (Figure 5C, Table S2). Both anti-biofilm assay and anti-hyphal assay results indicated that *meta*-dimethoxy was very important for the anti-biofilm formation activity. 

## 3. Discussion and Conclusion

In this study, we evaluated the SAR of PTE analogues against *C. albicans* biofilms.The structure of PTE was divided into three partitions: partition A contains 4-hydroxy benzene, partition B is a double bond, and partition C contains 3,5-dimethoxy benzene. By comparing a series of compounds including PTE, we found that, in partition A, *para*-hydroxy (–OH) took advantage over other substituents, and the double bond in partition B and the *meta*-dimethoxy (–OCH_3_) in partition C contributed to the best activity. 

Regarding partition A, once hydroxy (–OH) was replaced by other substituents, the activity against biofilms disappeared. It could be inferred that *para*-hydroxy (–OH) was important for the activity of PTE analogues. Regarding partition B, when the double bond was changed to a single bond (C6), the activity became weakened, indicating that the double bond contributed to the best activity. Interestingly, when the double bond became a part of a furan ring (B3), the compound showed almost the same inhibitory activity against biofilms as PTE. However, when the double bond became a part of an unsaturated lactone (C7), or partition A and partition B formed cyclic ester (B4), the activity was almost lost. We analyzed the structure of the compounds, and found that the double bond in PTE and B3 might help them to maintain the planarity. In contrast, in C7 and B4, although the double bond still existed, the formed ester might disrupt the planarity of the structure. Thereby, it could be inferred that the planarity in the center region of the compounds was helpful for their activities. Of note, we used E-type PTE to carry out all of the experiments because compounds in the Z-type are unstable. Moreover, Venera Cardilehad reported that *E*-stilbene was more active than its *Z*-isomer in most of the reported bioassays [12]. Regarding partition C, whether we added the number of methoxy (–OCH_3_) or changed methoxy (–OCH_3_) to hydroxy (–OH), the activity was weakened, suggesting that *meta*-dimethoxy (–OCH_3_) contributed to the best activity. Resveratrol (3,4’,5-trihydroxystilbene) is an analogue of PTE, and Francesco Carusohad tested the antifungal activity of resveratrol and its analogues against *Botrytis cinerea*, a facultative phytopathogenic fungus [13]. Notably, they also found that analogues with methoxy group (including PTE) exhibited better activity compared with resveratrol. Both our finding and Francesco Caruso’s suggested that the presence of methoxy groups might improve the antifungal activity of stilbene derivatives. However, previous findings also indicated that compounds with three methoxy groups were less active than the compounds with dimethoxy, because their demethoxylation was incomplete [13,14]. Methoxylation is an important molecular feature for membrane penetration, while subsequent demethoxylation may be necessary to make hydroxyls available for increasing antifungal activity [15]. Collectively, *meta*-dimethoxy (–OCH_3_) contributed to the best activity. In a word, SAR study in this work demonstrates that the activity of PTE analogues against biofilms is strongly associated with their structures.

We used XTT reduction assay to evaluate the anti-biofilm activity of the compounds, and anti-hyphal assay to confirm the results. Hyphae are widely known to be essential for maturely-developed biofilms [16]. The conclusion is supported by many findings. Firstly, hyphae are essential elements for providing the structural integrity and multilayered architecture characteristics of maturely developed biofilms [17]. Secondly, biofilms with a hyphal content of >50 % possessed significantly higher compressive strength and were more difficult to destroy by vortexing and sonication than biofilms with a lower hyphal content [18]. Thirdly, *C. albicans* deletion mutant with filamentation defect formed poor biofilms, lacking a three-dimensional structure and composed mainly of sparse monolayers of elongated cells [19]. In accordance, consistent results were obtained through biofilm experiments and hypha-inducing experiments. Moreover, the results obtained by anti-hyphal assay really confirmed the findings through anti-biofilm assays in this work.

Although the results obtained by anti-biofilm assay and anti-hyphal assay were consistent in general, compounds were easier to exhibit anti-hyphal activity in Spider medium compared with RPMI 1640 medium. In accordance, Karla J. Daniels found that biofilms formed in RPMI 1640 medium and Spider medium differed in cellular architecture, matrix deposition, penetrability by leukocytes, fluconazole susceptibility, and the facilitation of mating [20]. Moreover, RPMI 1640 and Spider media might activate different regulatory pathways [20,21]. From the findings in this study, the hyphal formation in RPMI 1640 medium was relatively more difficult to inhibit. Nevertheless, PTE, and its analogues C4, C6, B3, B5, and B6, inhibited hyphal formation in a concentration-dependent manner in all the media tested.

In conclusion, *para*-hydroxy (–OH) takes advantages over other substituents, double bonds, and *meta*-dimethoxy (–OCH_3_) contribute to the best activity of PTE against *C. albicans* biofilms. These findings may aid the development of drugs for biofilm treatment.

## 4. Materials and Methods

### 4.1. Strains, Culture, and Agents

*C. albicans* strain SC5314 is a most widely used clinical strain in the *Candida* research field [22,23] and was used in this study. SC5314 was routinely grown in YPD (1% yeast extract, 2% peptone, and 2% dextrose) liquid medium at 30 °C in a shaking incubator [24]. PTE and other analogues were synthesized by ZhongshanWanYuan New Drug R & D Co., Ltd. (Zhongshan, China) [25,26,27]. The level of purity of the synthesized analogues is ≥99% by HPLC (ZhongshanWanYuan New Drug R & D Co., Ltd., Zhongshan, China). For in vitro experiments, 6.4 mg/mL agents in DMSO were used as a stock and added to the culture suspensions to obtain the required concentrations. Other media included Spider and RPMI 1640 medium [28].

### 4.2. In vitro Biofilm Formation Assay

The assay was performed according to the method described previously [29] with slight modifications. Briefly, biofilm formation assay was performed in a 96-well tissue culture plate (Corning, cat. No. 3599) by seeding 100 μL cell suspension (1.0 × 10^6^ cells/mL) in selected wells in RPMI 1640 medium (Gibco, Bethesda, MD, USA), and incubating them statically at 37 °C. After 90-min adhesion, the medium was discarded, non-adherent cells were removed, and fresh RPMI 1640 medium was added. To detect the effect of different analogues on the formation of biofilms, different concentrations of analogues were added to the fresh RPMI 1640 medium after 90-min adhesion, and incubated at 37 °C for 24 h. To detect the effect of PTE and analogues on mature biofilms, *C. albicans* biofilms were formed at 37 °C for 24 h as described above. The biofilm supernatant was then discarded and fresh RPMI 1640 medium containing different concentrations of PTE or analogues was added. The plate was incubated at 37 °C for additional 24 h to observe the anti-biofilm effect [30]. A semiquantitative measure of the formed biofilms was calculated using an XTT reduction assay [31]. Each analogue was tested in triplicate. SMIC_80_ were determined as the lowest concentration of the compound that inhibited biofilm formation by 80%.

### 4.3. Hyphal Formation Assay

Hyphal formation assay was carried out to determine the ability of different analogues to inhibit *C. albicans* yeast-to-hyphal transition [32]. Briefly, *C. albicans* cells were harvested and washed with PBS. Spider and RPMI 1640 liquid media were used seperately in the following hyphal-inducing experiment. 1 × 10^6^ cells/mL *C. albicans* were suspended in hyphal-inducing medium. Cell suspensions with different concentrations of compounds were added to 12-well plates. The plates were incubated at 37 °C for 3 h, and photos were taken on the AMG EVOS x 1 (Amersham Pharmacia, Pittsburgh, PA, USA).

## Figures and Tables

**Table 1:** Structure and SMIC_80_ of PTE and its analogues.

Name	Structure	SMIC_80_ (μg/mL)	
		Biofilm Formation	Mature Biofilm
PTE	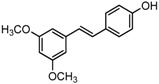	16	128
C4	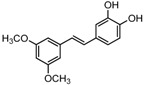	32–64	512
C1	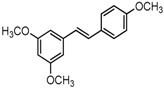	>64	>512
C2	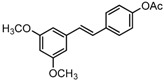	>64	>512
C3	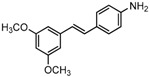	>64	>512
B1	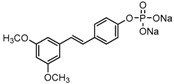	>64	>512
B2	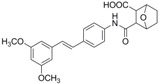	>64	>512
C5	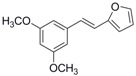	>64	>512
C6	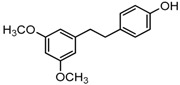	32-64	512
B3	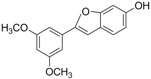	32	128–256
C7	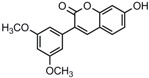	>64	>512
B4	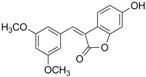	>64	>512
B6	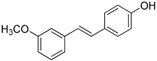	64	>512
B5	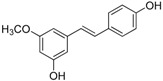	>64	>512
C8	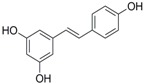	>64	>512
C9	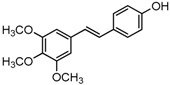	>64	>512

## Supplementary Materials

Supplementary materials are available online.
