# Supplementary material for: The Structure-Activity Relationship of Pterostilbene Against Candida albicans Biofilms
Source: Molecules. 2017 Feb 27;22(3):360. doi: 10.3390/molecules22030360 (PMC6155180; doi:10.3390/molecules22030360)
Supplement: Supplementary file 1 [file molecules-22-00360-s001.pdf]

Table S1. SMIC80 and structure of the analogues of PTE.

Name	Structure	SMIC$_{80}$ (μg/ml)	
		**Biofilm formation**	**Mature biofilm**
C11	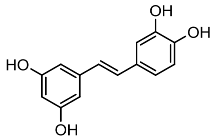	>64	>512
C12	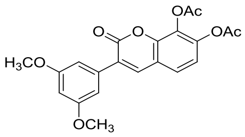	>64	>512
C13	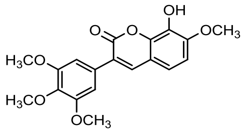	>64	>512
C14	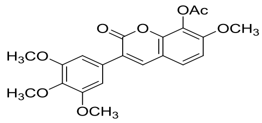	>64	>512
C15	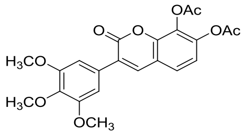	>64	>512
C16	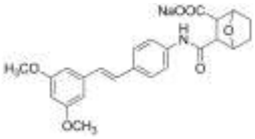	>64	>512
C17	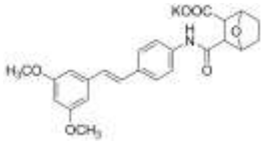	>64	>512
C18	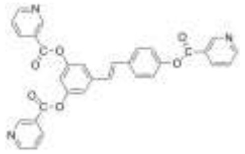	>64	>512
C19	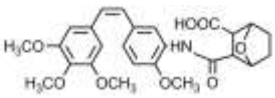	>64	>512
C10	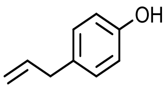	>64	>512

C20	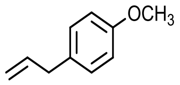	>64	>512
C21	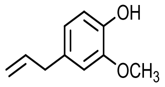	>64	>512
C22	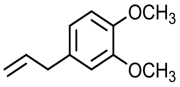	>64	>512

Table S2. The rate of hyphal formation after treatment of PTE and analogues.

**Name**	**RPMI 1640**						**Spider**					
	**0**	**4**	**8**	**16**	**32**	**64**	**0**	**4**	**8**	**16**	**32**	**64**
**PTE**	99.7	91	49.3	0	0	0	97	82.6	32.5	0	0	0
**C4**	99.7	84.2	62.2	50.1	1	0	97	77.3	54.3	17.9	0	0
**C1**	99.7	98.7	92	90	83.1	80.5	97	97.2	95	89.5	85	83.6
**C2**	99.7	95	90.3	91	77.7	35.7	97	34.7	13	10.3	12.7	5.7
**C3**	99.7	93	83.4	74.8	59.3	47.5	97	43.3	25.7	15.7	13	7
**B1**	99.7	99	100	97	97.3	94	97	100	98.7	96	91.2	87
**B2**	99.7	98	90.9	94.1	97.3	77.1	97	100	98.5	97.3	93.6	89
**C5**	99.7	97	95.6	91.6	86.1	83	97	89.4	80.7	54.4	39.5	6
**C6**	99.7	95.4	86.1	48.7	15	0	97	12.5	10	2	0	0
**B3**	99.7	94.5	79	20.5	3	0	97	23.3	5	0	0	0
**C7**	99.7	88.9	82.5	35.6	32.7	4	97	57.9	40.5	35	33.7	13
**B4**	99.7	100	94.2	90	91.7	88	97	93	95.9	92	89.2	84
**B6**	99.7	78.3	70.9	22	0	0	97	15	11	3	0	0
**B5**	99.7	98.3	90	68.8	29	13	97	93	89.2	51.2	16	1
**C8**	99.7	98.6	94.5	90.6	87.9	83.1	97	99.3	94.4	95	90.1	80.5
**C9**	99.7	99.7	100	89.4	83.2	79.1	97	86.4	65.1	71.3	67.2	37.2

1. The rate of hyphal formation (%) = (the number of cells with hyphal ≥40 μm/100)*100

2. Concentration: μg/ml
